# Relationship of Bacterial Richness to Organic Degradation Rate and Sediment Age in Subseafloor Sediment

**DOI:** 10.1128/AEM.00809-16

**Published:** 2016-07-29

**Authors:** Emily A. Walsh, John B. Kirkpatrick, Robert Pockalny, Justine Sauvage, Arthur J. Spivack, Richard W. Murray, Mitchell L. Sogin, Steven D'Hondt

**Affiliations:** a Graduate School of Oceanography, University of Rhode Island, Narragansett Bay Campus, Narragansett, Rhode Island, USA; b Department of Earth and Environment, Boston University, Boston, Massachusetts, USA; c Josephine Bay Paul Center for Comparative Molecular Biology and Evolution, Marine Biological Laboratory, Woods Hole, Massachusetts, USA; Stanford University

## Abstract

Subseafloor sediment hosts a large, taxonomically rich, and metabolically diverse microbial ecosystem. However, the factors that control microbial diversity in subseafloor sediment have rarely been explored. Here, we show that bacterial richness varies with organic degradation rate and sediment age. At three open-ocean sites (in the Bering Sea and equatorial Pacific) and one continental margin site (Indian Ocean), richness decreases exponentially with increasing sediment depth. The rate of decrease in richness with increasing depth varies from site to site. The vertical succession of predominant terminal electron acceptors correlates with abundance-weighted community composition but does not drive the vertical decrease in richness. Vertical patterns of richness at the open-ocean sites closely match organic degradation rates; both properties are highest near the seafloor and decline together as sediment depth increases. This relationship suggests that (i) total catabolic activity and/or electron donor diversity exerts a primary influence on bacterial richness in marine sediment and (ii) many bacterial taxa that are poorly adapted for subseafloor sedimentary conditions are degraded in the geologically young sediment, where respiration rates are high. Richness consistently takes a few hundred thousand years to decline from near-seafloor values to much lower values in deep anoxic subseafloor sediment, regardless of sedimentation rate, predominant terminal electron acceptor, or oceanographic context.

**IMPORTANCE** Subseafloor sediment provides a wonderful opportunity to investigate the drivers of microbial diversity in communities that may have been isolated for millions of years. Our paper shows the impact of *in situ* conditions on bacterial community structure in subseafloor sediment. Specifically, it shows that bacterial richness in subseafloor sediment declines exponentially with sediment age, and in parallel with organic-fueled oxidation rate. This result suggests that subseafloor diversity ultimately depends on electron donor diversity and/or total community respiration. This work studied how and why biological richness changes over time in the extraordinary ecosystem of subseafloor sediment.

## INTRODUCTION

Subseafloor sediment contains a diverse microbial ecosystem (1–3), with a total cell abundance comparable to that in terrestrial soil and in the world ocean (4). Subseafloor sedimentary communities push the boundaries of life as we know it; per-cell rates of respiration are often orders of magnitude lower than those in the surface world (5, 6), biomass turnover can take hundreds to thousands of years (7, 8), cell abundance can be as low as 10 cells per cm^3^ (9), and microbes in deep subseafloor sediment may be isolated from the surface world for millions of years (Ma) to tens of Ma. Subseafloor sediment, therefore, provides an unprecedented opportunity to investigate drivers of microbial diversity on a time scale of thousands to millions of years.

In the broadest context, distributions of microbial diversity result from combined effects of speciation, selection, dispersal, and ecological drift (10, 11). However, subseafloor conditions may severely impact the relative influence of these processes. For example, exceedingly low per-cell energy fluxes may place very high selection pressure on subseafloor populations, severely limit active dispersal (6) and cell abundance, and cause mean generation times to greatly exceed the already-long few-hundred-year to few-thousand-year time scale of biomass turnover (7) in subseafloor sediment (12). Generation times of hundreds to millions of years may in turn greatly lower the rates of speciation.

**FIG 1:**
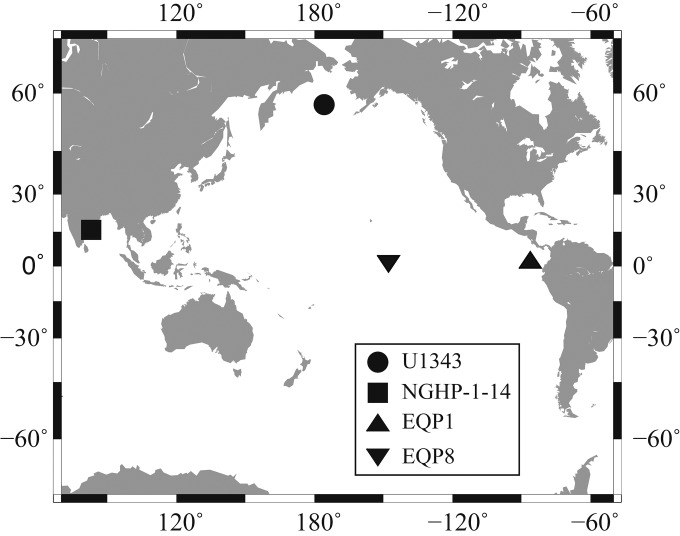
Sampling locations. (Map created with Generic Mapping Tools.)

To document microbial diversity and its potential drivers in subseafloor sediment, we extracted and sequenced PCR amplicons for the V4 to V6 hypervariable region of the bacterial 16S rRNA gene from the sediment of four distinct locations: the Bering Sea (Integrated Ocean Drilling Program [IODP] expedition 323 site U1343) (13), the eastern equatorial Pacific (*Knorr* expedition 195-3 site EQP1), the central equatorial Pacific Ocean (*Knorr* 195-3 site EQP8), and the Bay of Bengal continental margin (Indian National Gas Hydrate Program [NGHP] site NGHP-1-14) (14) (Fig. 1).

## MATERIALS AND METHODS

### Sites.

The three open-ocean sites (Bering Sea site U1343 and Equatorial Pacific sites EQP1 and EQP8) have water depths of 1,953, 2,885, and 4,336 m below sea level (mbsl), respectively (see the supplemental material). The water depth at the Bay of Bengal continental margin site NGHP-1-14 is 895 mbsl (14). The Bering Sea and Bay of Bengal sites are characterized by high sea surface chlorophyll concentrations and very high sedimentation rates (0.34 and 1.04 mg/m^3^ and 250 m/Ma, and ca. 100 m/Ma at U1343 and NGHP-1-14, respectively). The equatorial Pacific sites are characterized by moderate sea surface chlorophyll concentrations and moderate mean sedimentation rates (0.16 and 0.32 mg/m^3^, and 75 and 4.8 m/Ma at EQP1 and EQP8, respectively). The total organic carbon (TOC) content of near-surface sediment is highest at the high sedimentation sites (0.6% and 1.7% at NGHP-1-14 and U1343, respectively) and lowest at the moderate sedimentation sites (0.1 and 0.02% at EQP1 and EQP8, respectively). The maximum sampled sediment depths range from 27 m below seafloor (mbsf) at EQP8 to 404 mbsf at U1343 (see Table S1 in the supplemental material). The concentration profiles of the dissolved metabolic products and substrates (dissolved inorganic carbon [DIC], methane, ammonium, oxygen, nitrate, and sulfate) indicate that microbial activity occurs throughout the sampled sequences (15).

### Shipboard sampling and geochemistry.

Immediately after core recovery, we cleaned the cut face of the remaining core section with a sterile blade. For DNA analysis of NGHP-1-14, we cut 10-cm whole-core rounds from the core sections. For EQP1, EQP8, and U1343, we took samples for DNA analysis from the center of the cleaned core face using sterile 60-cm^3^ cutoff syringes. We froze the samples at −80°C for shore-based DNA analysis. Concentrations of DIC, sulfate, and, for site U1343, methane were measured during the expeditions, according to standard procedures (2, 13, 14, 16). We made TOC measurements, as previously described (17), on a Costech elemental analyzer. All geochemical and environmental data for site U1343 are deposited in the IODP database and accessible online in the IODP expedition 323 proceedings (13), except for the postcruise TOC (see the supplemental material). All dissolved geochemical measurements for sites EQP1, EQP8, and NGHP-1-14 are deposited at EarthChem (www.earthchem.org). The cell count data for EQP1, EQP8, and U1343 are available in a study by Kallmeyer et al. (4). The chlorophyll data are from a study by Gregg (18). We used the method of Sauvage et al. (19) to account for changes in DIC and alkalinity that resulted from the precipitation of carbonate during the recovery and processing of core samples from EQP1, EQP8, and U1343 (see the supplemental material).

### Pyrosequencing, clustering, and diversity analyses.

We extracted DNA from the sediment samples using commercial kits (MoBio PowerSoil). We amplified the V4 to V6 hypervariable region of the 16S rRNA gene using the bacterial primer pair 518f-1064r and pyrosequenced the amplicons according to standard protocols on a 454 GS-FLX sequencer at the Josephine Bay Paul Center, Marine Biological Laboratory, Woods Hole, MA (20). To reduce error, we removed low-quality sequences (such as those with low average quality scores or deviations in read length) prior to analysis, as described in Huse et al. (21). Sequencing protocols, analyses, and initial results are accessible at the VAMPS website (https://vamps.mbl.edu/index.php) under the projects DCO_WAL_Bv6v4, KCK_EQP_Bv6v4, and JBK_IO_Bv6v4. For further analysis, we determined the taxonomy of each sample at the genus level using the SILVA database (22) on the VAMPS website. From EQP1 samples, we removed all sequences that correspond to Vibrio, because it was actively cultured in the laboratory used for EQP1 DNA extraction. No Vibrio DNA occurs in samples from the other sites, which were extracted in a different dedicated laboratory.

We used QIIME (23), as made available on N3phele (24), to cluster each sample into operational taxonomic units (OTUs) at the 3% similarity-level. To remove the effects of sampling intensity (number of reads) on downhole or intersite comparisons of richness estimates (25), we first randomly subsampled the number of reads in each sample to the lowest number found in any sample (*n* = 2,800). OTUs were picked from subsampled sequences using Uclust (26) with the furthest neighbor approach. Representative sequences for each OTU were assigned RDP taxonomy (27), aligned with PyNAST (28), and a distance matrix was calculated using UniFrac (29). Clustering average-neighbor OTUs with mothur's MiSeq standard operating procedures (SOP) (30, 31) identified more OTUs than with QIIME but exhibited similar trends of richness with sediment depth and age. To investigate patterns of diversity with changes in depth, we also clustered samples at multiple levels of similarity to generate comparisons between different similarity cutoff levels (see the supplemental material).

We used the distance matrix and OTU tables created with QIIME for statistical analyses of diversity. We calculated the Chao1 index (25) using QIIME. We compared these results to richness metrics calculated with CatchAll (32) (see the supplemental material). We performed Bray-Curtis similarity analyses, nonmetric multidimensional scaling, and Spearman rank correlation tests using the Primer 6 program (33).

### Sediment age calculations.

We calculated sediment age estimates for U1343 using the sediment age model of Takahashi et al. (13). The U1343 age model is based on biostratigraphic and magnetostratigraphic data (13). No detailed chronostratigraphic data are available for EQP1 or EQP8; consequently, we estimated their sediment ages from their average sedimentation rates (sediment thickness [50] divided by basement age [41]). Because no published chronostratigraphic data are available for NGHP-1-14, our age model for that site is based on the biostratigraphically determined sedimentation rates of other NGHP sites in the same basin (ca. 110 m/Ma and ca. 125 m/Ma at NGHP-1-16 and NGHP-1-10, respectively [42]). The shallowest NGHP-1-14 samples may be younger than our age estimates, since relatively shallow vertical variation in its dissolved chemical profiles may have resulted from the deposition of ca. 13 m of sediment by a mass transport event about 1,400 years ago (34).

### Reaction rate calculations.

To quantify the net rates of organic-fueled respiration from dissolved chemical data of EQP1, EQP8, and U1343, we used a modified version of the Matlab-based numerical procedures of Wang et al. (35). Similar calculations are not possible for continental margin site NGHP-1-14, because its dissolved chemical concentration profiles are not in diffusive steady state. We modified the approach of Wang et al. (35) by using an Akima spline, instead of a 5-point running mean, in order to generate a best-fit line to the chemical concentration data. We determined standard deviations through use of a Monte Carlo simulation (*n* = 30). For EQP1, EQP8, and U1343, we calculated organic-fueled respiration from DIC concentration profiles after first correcting DIC and alkalinity concentrations to account for carbonate precipitation during sediment recovery, processing, and storage (19). For U1343, we also calculated net organic-fueled respiration from the ammonium concentration profile to independently check the organic-fueled respiration rates calculated from DIC concentrations.

## RESULTS

**FIG 2:**
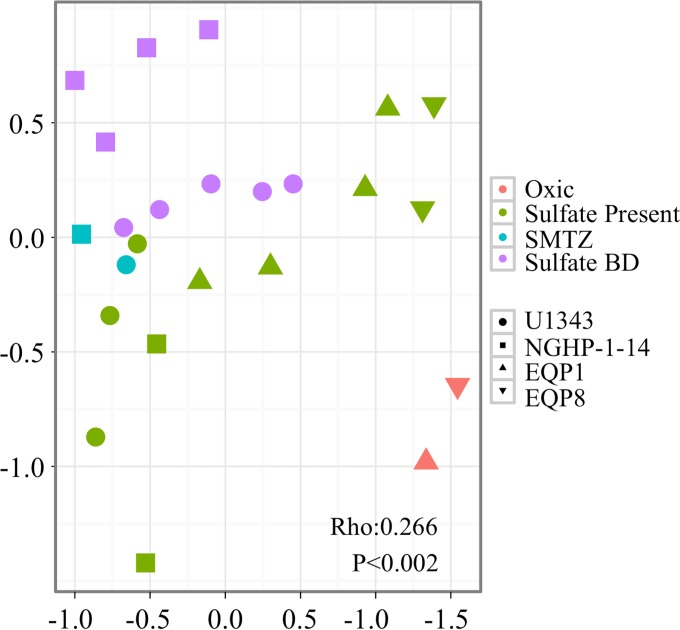
Nonmetric multidimensional scaling (nMDS) plot. Bray-Curtis distances between samples represent the degree of community similarity (samples that contain similar communities are closer together in ordination space [stress = 0.01]). Symbol color indicates redox zone, and symbol shape indicates site location as shown in Fig. 1.

Abundance-weighted community composition broadly varies with the vertical succession of chemical redox zones (36, 37), with Bray-Curtis similarity scores exhibiting a clear gradient through (i) the oxygenated zone immediately beneath the seafloor, (ii) a deeper anoxic zone with abundant dissolved sulfate, (iii) a still-deeper sulfate/methane transition zone (SMTZ), and (iv) the deepest zone, with little or no sulfate but abundant dissolved methane (Fig. 2). This result expands on earlier discoveries that dominant microbial taxa in the SMTZ or in subseafloor hydrates differ from the dominant taxa in overlying or underlying sediment (38–40).

**FIG 3:**
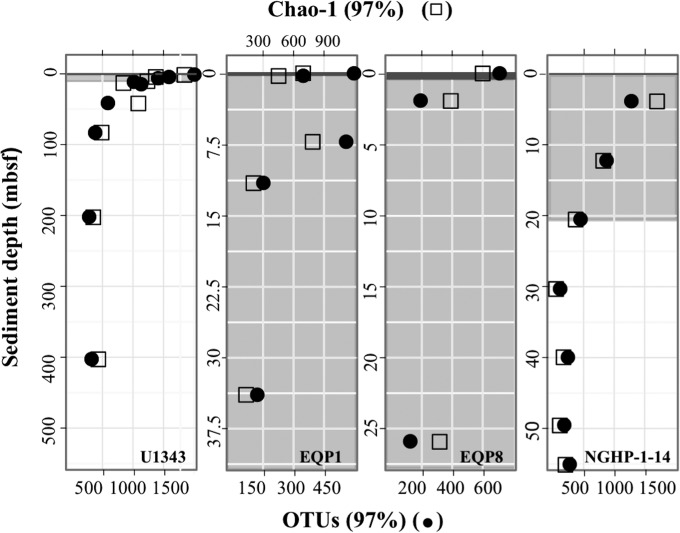
Comparison of OTU richness to redox zones. Filled circles identify numbers of OTUs. Open squares show Chao1 richness values. Dark gray bar indicates oxygen penetration depth, light gray grid indicates the presence of sulfate, and white background indicates sulfate is below detection levels.

In contrast to abundance-weighted community composition, total bacterial diversity does not demonstrate a vertical succession of redox zones. Bacterial taxonomic richness, as measured by both Chao1 estimates and numbers of operational taxonomic units (OTUs) in samples normalized to equal numbers of reads, is highest near the seafloor and drops exponentially with increasing sediment depth at all four sites (Fig. 3). The trend is the same for parametric analyses (CatchAll [32]) (see the supplemental material). The rate of decrease in richness with depth varies greatly from site to site; OTU richness approaches low relatively stable values several tens of meters below the seafloor at site U1343 but within a couple of meters below seafloor at site EQP8. This exponential decline in richness occurs at every phylogenetic level: whether defined by similarity as high as 100% or as low as 85%, the number of operational taxonomic units in normalized samples declines with increasing sediment depth (see the supplemental material).

The rate of decrease in OTU richness is not clearly associated with any particular geochemical zone or transition between zones. The inflection from rapidly declining richness to relatively stable richness occurs within sulfate-replete sediment at EQP1 and EQP8, at or near the SMTZ at NGHP-1-14, and within the methane-rich sulfate-poor zone at site U1343.

The bacterial richness of our shallowest samples varied considerably from site to site, with richness of 97%-similar OTUs ranging from 1,951 at U1343 to 572 at EQP1 (Fig. 3). This variation is not surprising, because there are large environmental differences between sites (they underlie different oceanographic regimes and sample very different kinds of sediment), and because the shallowest samples differ greatly in sediment age (ranging from a few hundred years at U1343 to ∼10,000 years at EQP8). At greater depths, OTU richness at all sites decreases to values in a similar range (150 to 300 OTUs).

**FIG 4:**
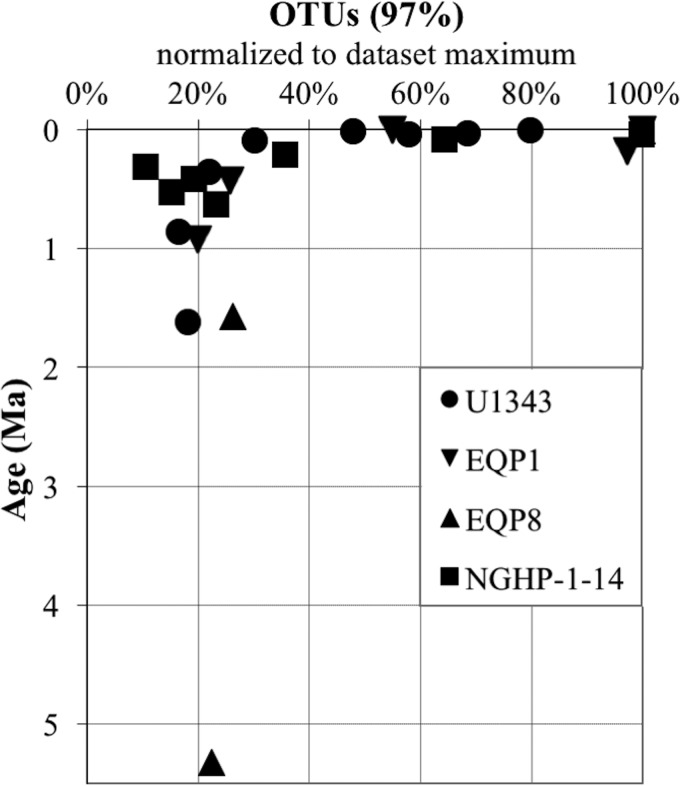
OTU richness relative to sediment age. To normalize OTU counts between sites, we set the most OTU-rich sample for each site (always the shallowest sample, in the upper right corner) to 100% and calculated the richness of deeper samples calculated as percentages of that number. Symbol shape indicates site location.

The transformation of sediment depth to sediment age shows that OTU richness decreases exponentially with age at all four sites (Fig. 4). It decreases rapidly in the youngest sediment and then stabilizes or decreases more slowly with greater age. Despite the large site-to-site differences in sedimentation rate, oceanographic context, and predominant electron acceptor regimes, OTU richness consistently takes a few hundred thousand years to decline from near-seafloor sediment levels to much lower values in deep-subseafloor sediment.

**FIG 5:**
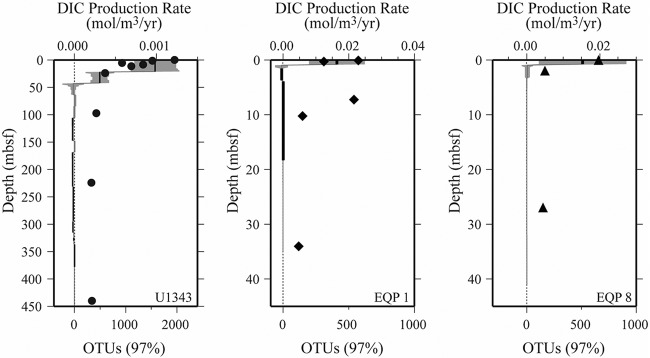
Relationship of OTU richness to organic respiration rate (as indicated by DIC production) at the open-ocean sites (U1343, EQP1, and EQP8). Black symbols indicate numbers of OTUs. Black lines and gray bars indicate reaction rates and two times the standard deviation, respectively. The data are plotted against sediment depth (mbsf) for each site.

Vertical patterns of richness covary with DIC production rates at open-ocean sites U1343, EQP1, and EQP8 (Fig. 5). At EQP1 and EQP8, DIC production neatly represents gross organic-fueled respiration; the cored sequence is rich in external electron acceptors (nitrate and sulfate) and there is no evidence of major DIC sinks in the cored sediment (e.g., dissolved calcium or magnesium sinks indicating carbonate precipitation). The situation is slightly more complicated at U1343, where (i) the DIC profile is slightly modified by net DIC consumption (carbonate precipitation) in some intervals at depths greater than 100 mbsf (15) (Fig. 4) and (ii) external electron acceptors are scarce below the sulfate-methane interface at ∼8 mbsf (13). A comparison of the U1343 DIC production rates to net ammonium production rates demonstrates that these modifications are of secondary importance, because the calculated profile of DIC production broadly matches the vertical profile of organic-fueled degradation estimated from ammonium production (see the supplemental material).

At all three sites (U1343, EQP1, and EQP8), the rate of organic degradation indicated by net production of DIC and ammonium is highest near the seafloor, where OTU richness is also highest (Fig. 5). At U1343, organic degradation, like OTU richness, takes tens of meters to decline to extremely low values. At EQP1 and EQP8, the large declines in both organic degradation and OTU richness occur within the first few meters below the seafloor.

## DISCUSSION

The relationships between abundance-weighted community composition and redox zones (Fig. 2) indicate that some taxa are influenced by the predominant terminal electron-accepting activity. In contrast, the lack of clear correspondence between bacterial richness and redox zonation suggests that the predominant terminal electron-accepting pathway does not exert primary control on OTU richness of subseafloor bacterial communities. Possible explanations of this lack of relationship between OTU richness and predominant terminal electron acceptors include the following: (i) most OTUs may represent taxa that are not involved in terminal electron acceptance and that operate similarly in successive redox zones (for example, fermentative taxa may be active in all of the anoxic zones), (ii) taxa directly involved in terminal electron acceptance may be capable of processing multiple kinds of electron acceptors, and (iii) terminal electron acceptance may not be limited to the predominant pathway, with terminal electron-accepting activity predominant in one redox zone also existing in other zones (for example, iron reducers may be present and active where sulfate reduction, methanogenesis, or sulfide oxidation predominate [51, 52, 53]).

The exponential decline in bacterial richness from seafloor to greater sediment depth is consistent with recent comparisons of bacterial OTUs in the ocean to OTUs in marine sediment (43). Based on the relative abundance of 16S V6 tags in the water column, shallow sediment (0 to 0.1 mbsf), and subseafloor sediment, these studies show that (i) marine sedimentary bacteria are dispersed via the ocean, and (ii) subseafloor sedimentary lineages are selected from the community present in shallow sediment (43).

The close match between the exponential decline in bacterial richness and the depth distribution of organic degradation rates at our open-ocean sites indicates that vertical variation in richness is closely tied to organic-fueled community activity. The pattern of organic degradation exponentially declining from seafloor to greater sediment depth was first observed decades ago. It is often explained with a “multi-G model,” in which organic matter is assumed to be composed of diverse organic compounds with different levels of reactivity (44). In such models, the most labile or biologically reactive organic substrates are respired at much higher rates than the least-labile substrates, leading the net rates of organic-fueled respiration to decrease exponentially with increasing sediment depth (44, 45).

A single EQP1 sample at 7.21 mbsf constitutes the only exception to the close correspondence between exponentially declining richness and exponentially declining organic degradation at these sites. This relatively OTU-rich sample contains an unusually high concentration of organic matter relative to adjacent sediment; its exceptionality is consistent with previous research that showed that discrete horizons of organic-rich sediment may sustain locally high respiration for millions of years (46).

Near continental shelves (such as the Indian Margin) and in upwelling regions (such as the Bering Sea and the equatorial Pacific), organic matter is the primary electron donor for subseafloor sedimentary communities (2). Consequently, the close correspondence between vertical patterns of richness and vertical patterns of organic degradation suggests that selection for organismal properties related to either total catabolic activity or electron donor diversity exerts the primary influence on bacterial OTU richness in anoxic subseafloor sediment. This correspondence also indicates that many bacterial taxa that are poorly adapted for subseafloor sedimentary conditions are degraded in the geologically young sediment where respiration rates are high.

This result sets a clear boundary for understanding bacterial OTU richness in anoxic subseafloor sediment. It also provides a potential basis for ultimately integrating OTU richness with other key properties that appear to be broadly related to total catabolic activity in subseafloor sedimentary communities, such as cell (4) or viral particle abundance (47) and activity (48). However, the exact traits that preferentially aid survival as catabolic activity and/or electron donor diversity decline remain to be determined; candidate traits include specialization to metabolize recalcitrant organic substrates, specific energy-conserving properties, such as membrane permeability (6), use of sodium ions for energy storage (6), spore formation (6), prophage modulation of metabolic activity (49), and/or a wide range of other properties (12).

## Supplementary Material

Supplemental material
