# Supplementary material for: Relationship of Bacterial Richness to Organic Degradation Rate and Sediment Age in Subseafloor Sediment
Source: Appl Environ Microbiol. 2016 Jul 29;82(16):4994–9. doi: 10.1128/AEM.00809-16 (PMC4968545; doi:10.1128/AEM.00809-16)
Supplement: Supplemental material [file supp_82_16_4994__index.html]

Supplemental material 

# Relationship of Bacterial Richness to Organic Degradation Rate and Sediment Age in Subseafloor Sediment

## Supplemental material

- Supplemental file 1 -

  Supplemental site data and results, environmental and sampling data (Table S1), plots of bacterial richness at multiple levels of genetic similarity (Fig. S1), Comparison of richness values calculated using the best parametric model as calculated by CatchAll to values calculated using the Chao1 metric (Fig. S2), vertical distributions of richness values (Fig. S3), taxonomic (phylum-level) breakdown of dominant taxa (Fig. S4), vertical profiles of measured DIC values and corrected DIC values (Fig. S5), and vertical profiles of organic carbon oxidation (Fig. S6).

  PDF, 2.2M
