# Supplementary material for: Relationship of Bacterial Richness to Organic Degradation Rate and Sediment Age in Subseafloor Sediment
Source: Appl Environ Microbiol. 2016 Jul 29;82(16):4994–9. doi: 10.1128/AEM.00809-16 (PMC4968545; doi:10.1128/AEM.00809-16)
Supplement: Supplemental material [file AEM.00809-16_zam999117347so1.pdf]

Supplementary Information for

Relationship of bacterial richness to organic degradation rate and sediment age in subseafloor sediment

Emily A. Walsh[1], John B. Kirkpatrick[1], Robert Pockalny[1], Justine Sauvage[1], Arthur J. Spivack[1], Richard W. Murray[2], Mitchell L. Sogin[3] and Steven D'Hondt[1]

[1]Graduate School of Oceanography, University of Rhode Island, Narragansett Bay Campus, 215 South Ferry Road, Narragansett, RI 02882, USA

[2]Department of Earth and Environment, Boston University, Boston, MA 02066, USA

[3]Josephine Bay Paul Center for Comparative Molecular Biology and Evolution, Marine Biological Laboratory, 7 MBL Street, Woods Hole, MA 02543, USA

Site details

We present properties of each site in Table 1.

Richness of Operational Taxonomic Units at multiple cut-off levels

Bacterial taxonomic richness, as measured by numbers of operational taxonomic units (OTUs) in samples normalized to equal numbers of reads, is highest near the seafloor and drops exponentially with increasing sediment depth at all four sites. This exponential decline in richness occurs at every phylogenetic level; whether defined by similarity as high as 100% or as low as 85%, the number of operational taxonomic units in normalized samples declines with increasing sediment depth (Fig. S1).

Comparison of Chao1 richness estimates to Catchall parametric estimates

Because the Chao1 index may underestimate richness relative to parametric approaches, we compare our Chao1 estimates to estimates from parametric modeling of the diversity data using CatchAll[1]. The best Catchall parametric models for these data sets were evenly divided between two mixed exponential and three mixed exponential models; shown here are two mixed exponential results. In all cases, two mixed versus three mixed exponential model results were within one standard error of each other. Although our Catchall results vary somewhat from our Chao1 results in their absolute values of estimated richness, the vertical trends in richness are nearly identical for the two approaches (Figs. S2, S3). This analysis shows that the exponential declines in richness with sediment depth and age are real and not an artifact of the method used to calculate richness.

Taxonomic composition

We show the assigned taxonomy of dominant OTUs in figure S4. Many OTUs do not have well-defined taxonomy, either belonging to unknown members of candidate phyla or falling between known phyla of bacteria (Domain Bacteria; phyla NA). Dominant identifiable taxa include members of Chloroflexi (most notably Dehalococcoidetes), candidate division OP9, Planctomycetes, Deltaproteobacteria, Nitrospirae, and others.

Correction of dissolved inorganic carbon (DIC) values from EQP1 and EQP8

Because carbonate sediment is present in the sediment of EQP1 and EQP8, carbonate precipitation occurs in the sediment during the depressurization and warming associated with core recovery from the seafloor and subsequent sample processing. Such precipitation decreases DIC and alkalinity concentrations in the minutes and hours prior to porewater extraction. We used the method of Sauvage et al.[2] to account for changes in DIC and alkalinity that resulted from precipitation during recovery and processing of EQP1 and EQP8 core samples (Fig. S5).

Calculation of ammonium production and organic oxidation at U1343

For comparison of respiration calculated from the NH_4^+ profile to respiration calculated from the DIC profile at U1343 (Fig. S6), we converted NH_4^+ production to organic carbon oxidation by assuming that (i) all degraded organic matter had a Redfield C:N stoichiometry of 120:16 (following Anderson and Sarmiento[3]), (ii) all organic carbon degraded at depths shallower than 8 mbsf (the approximate depth of the sulfate-reducing methane oxidation front) was completely oxidized to CO_2, and (iii) only half of all organic carbon degraded at greater depths (where external electron acceptors are largely absent) is oxidized to CO_2 and the remaining organic matter degraded is in reduced form

(e.g., CH_4).

Supplemental References

1. **Bunge J**, **Woodard L**, **Böhning D**, **Foster J**, **Connolly S**, **Allen H**. 2012. Estimating population diversity with CatchAll. Bioinformatics **28**:1045–1047.
2. **Sauvage J**, **Spivack AJ**, **Murray RW**, **D'Hondt S**, 2014. Determination of in situ dissolved inorganic carbon concentration and alkalinity for marine sedimentary porewater. Chem. Geol. **387**:66–73.
3. **Anderson LA**, **Sarmiento JL**, 1995. Global ocean phosphate and oxygen simulations. Glob. Biogeochem. Cy. **9**:621-636.

Supplementary Table

VAMPS ID	Sample ID	Latitude	Longitude	Water Depth (m)	Sample Depth (mbsf)	Age (yrs)	TOC (wt%)	Sulfate (mM)	TON (wt%)	C:N Ratio	Cells (log10/g)
DCOWALBv6v4.Bering1Bv6v4	BS1	57°33.3993′N	175°48.9659′W	1953	0.16	615	1.14	25.85	0.129	8.83	7.6
DCOWALBv6v4.Bering2Bv6v4	BS2	57°33.3993′N	175°48.9659′W	1953	1.66	6384	0.84	20.85	0.104	8.13	6.2
DCOWALBv6v4.Bering3Bv6v4	BS3	57°33.3993′N	175°48.9659′W	1953	5.66	21768	0.93	11.88	0.105	8.91	6.2
DCOWALBv6v4.Bering4Bv6v4	BS4	57°33.3993′N	175°48.9659′W	1953	8.66	33306	0.80	0.00	0.223	3.56	5.7
DCOWALBv6v4.Bering5Bv6v4	BS5	57°33.3993′N	175°48.9659′W	1953	11.27	43344	0.66	0.00	0.089	7.41	5.4
DCOWALBv6v4.BS55B	BS5.5	57°33.3993′N	175°48.9659′W	1953	24.13	92804	0.70	0.00	0.110	6.31	5.3
DCOWALBv6v4.Bering6Bv6v4	BS6	57°33.3993′N	175°48.9659′W	1953	86.5	332679	0.59	0.00	0.097	6.06	4
DCOWALBv6v4.Bering7Bv6v4	BS7	57°33.3993′N	175°48.9659′W	1953	224	861504	0.61	0.00	0.099	6.17	2.5
DCOWALBv6v4.Bering8Bv6v4	BS8	57°33.3993′N	175°48.9659′W	1953	440	1692240	0.60	0.00	0.102	5.90	3.7
KCKEQPBv6v4.EQP1MC5cm	EQP 1 SEDA	01°48.2091'N	086°11.3787'W	2885	0.05	1351	0.59	27.64	0.092	6.46	9.4
KCKEQPBv6v4.EQP1MC25cm	EQP 1 SEDB	01°48.2091'N	086°11.3787'W	2885	0.25	6757	0.76	27.71	0.095	7.98	6.3
KCKEQPBv6v4.EQP1SEC4	EQP 1 SEDC	01°48.2091'N	086°11.3787'W	2885	7.21	194864	0.72	27.28	n/a	n/a	6.7
KCKEQPBv6v4.EQP1SEC6	EQP 1 SEDD	01°48.2091'N	086°11.3787'W	2885	10.18	275135	0.53	27.60	0.061	8.65	9.3
KCKEQPBv6v4.EQP134M	EQP 1 SEDE	01°48.2091'N	086°11.3787'W	2885	34	923513	0.44	24.87	0.068	6.49	7
KCKEQPBv6v4.EQP8SEDA	EQP 8 SEDA	00°00.5291'N	147°47.4947'W	4336	0.05	10081	0.33	27.82	0.038	8.69	8.9
KCKEQPBv6v4.EQP8SEDB	EQP 8 SEDB	00°00.5291'N	147°47.4947'W	4336	2.2	443548	0.28	27.89	0.020	14.12	8.0
KCKEQPBv6v4.EQP8SEDC	EQP 8 SEDC	00°00.5291'N	147°47.4947'W	4336	26	5241935	0.23	27.74	0.000	n/a	5.4
JBKIOBv6v4.NHGP14A4msediment	IN1	16° 03.5577' N	082° 05.6218' E	895	4	40000	1.42	17.14	0.095	14.85	n/a
JBKIOBv6v4.NHGP14A12msediment	IN2	16° 03.5577' N	082° 05.6218' E	895	12	89999	1.26	13.79	0.095	13.21	n/a
JBKIOBv6v4.NHGP14A21msediment	IN3	16° 03.5577' N	082° 05.6218' E	895	21	212220	1.47	4.59	0.095	15.41	n/a
JBKIOBv6v4.NHGP14A31msediment	IN4	16° 03.5577' N	082° 05.6218' E	895	31	317775	1.70	0.04	0.097	17.44	n/a
JBKIOBv6v4.NHGP14A40msediment	IN5	16° 03.5577' N	082° 05.6218' E	895	40	423329	1.46	0.04	0.102	14.29	n/a
JBKIOBv6v4.NHGP14A49msediment	IN6	16° 03.5577' N	082° 05.6218' E	895	49	528884	1.72	0.00	0.109	15.74	n/a
JBKIOBv6v4.NHGP14A59msediment	IN7	16° 03.5577' N	082° 05.6218' E	895	59	633105	0.97	0.00	0.076	12.71	n/a

Table S1. Environmental and sampling data for Sites U1343, EQP1, EQP8, and NGHP-14.

Supplementary Figures

Figure S1.

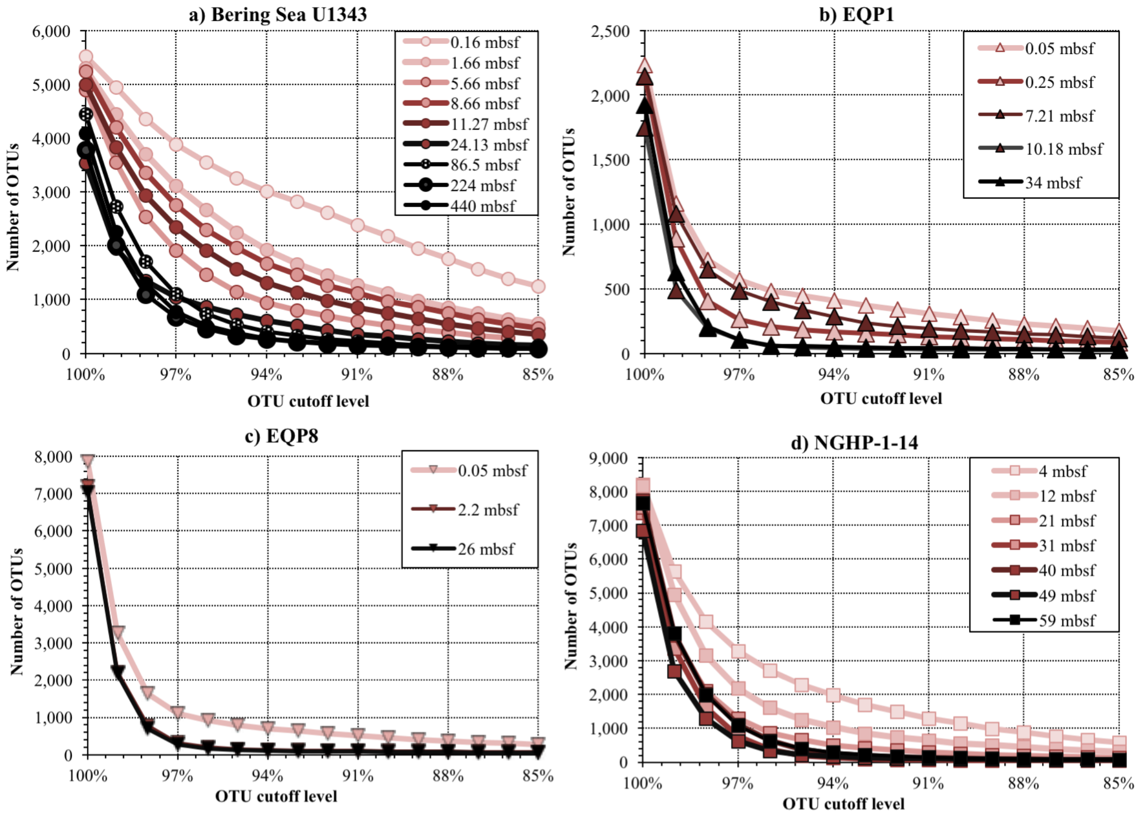

Figure S1. Plots of bacterial richness at multiple levels of genetic similarity, at each depth below seafloor: (A) U1343, (B) EQP1, (C) EQP8, and (D) NGHP-14. The x-axis indicates the cutoff level for clustering (percent sequence identity) and the y-axis indicates the number of OTUs generated. Sequence data at each site was subsampled to normalize each sample to the smallest number of reads for that site: (5,699 for U1343, 2,444 for EQP1, 9,174 for EQP8, and 9,159 for NGHP-14).

Figure S2.

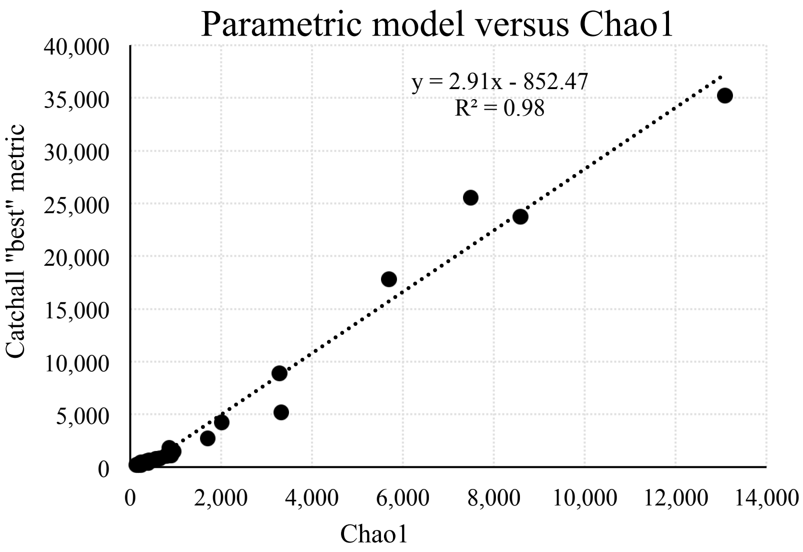

Figure S2. Comparison of (i) richness values calculated using the best parametric model as calculated by CatchAll (Bunge *et al.*[1]) to (ii) values calculated using the Chao1 metric.

Figure S3.

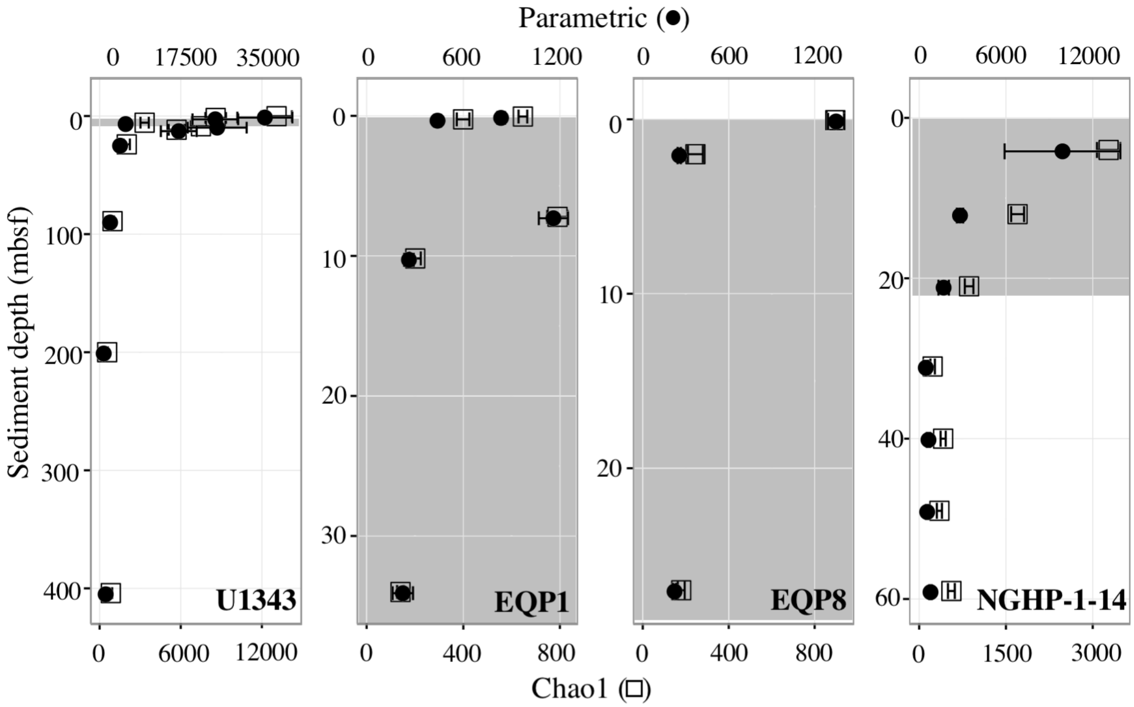

Figure S3. Vertical distributions of richness values calculated using (i) the bestparametric model as calculated with CatchAll (Bunge *et al.*[1]), (black circles) and (ii) the Chao1 diversity metric (white squares). Error bars indicate standard error. For black circles where no error bar is visible, standard error is smaller than the circle.

Figure S4.

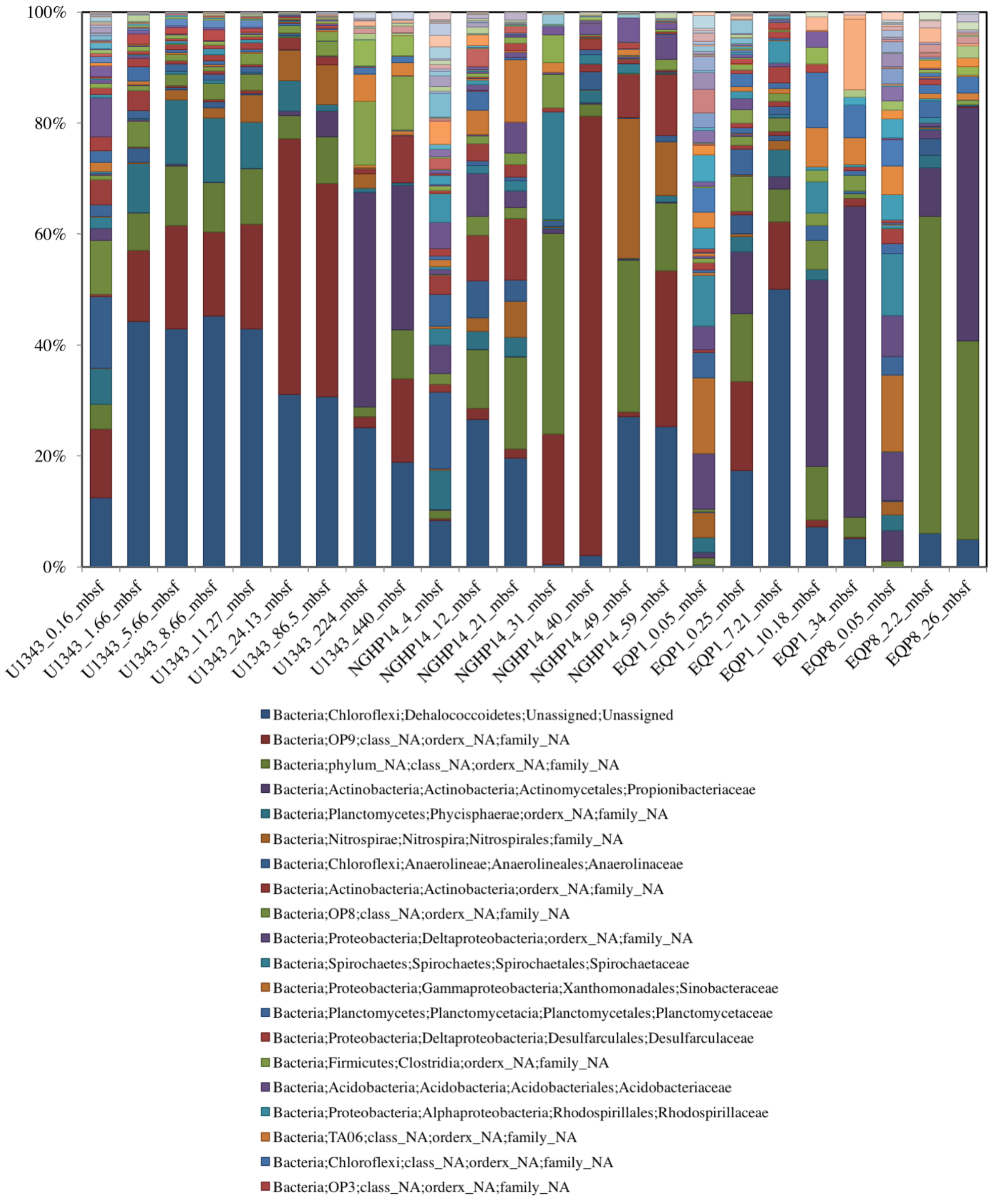

Figure S4. Taxonomic (phylum-level) breakdown of dominant taxa at U1343, NGHP-1-14, EQP1, and EQP8. For each site, samples are shown in depth order (mbsf = meters below seafloor).

Figure S5.

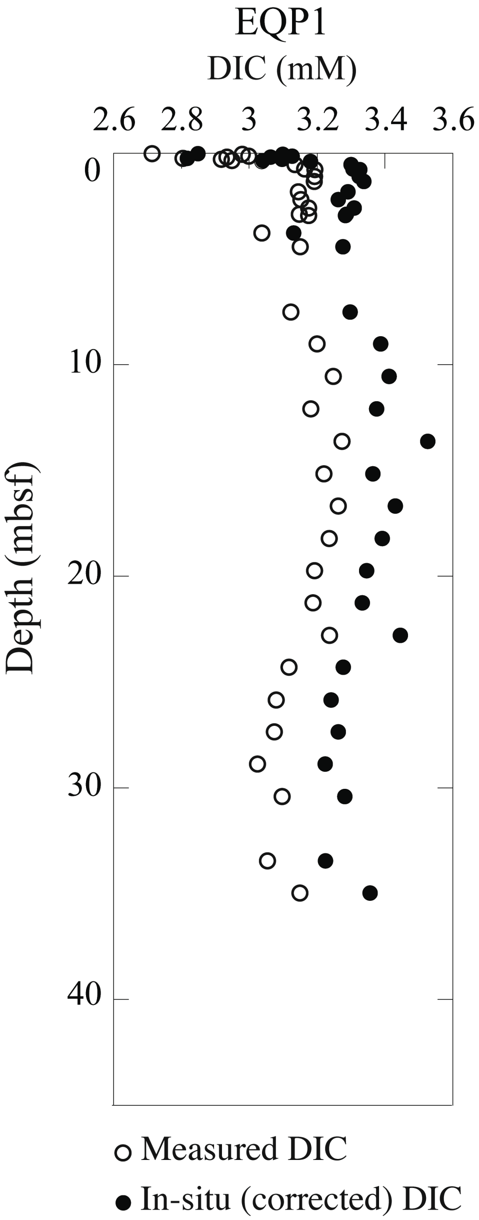

Figure S5. Vertical profiles of measured DIC values (red circles) and corrected DIC values (blue squares) at EQP1, and EQP8.

Figure S6.

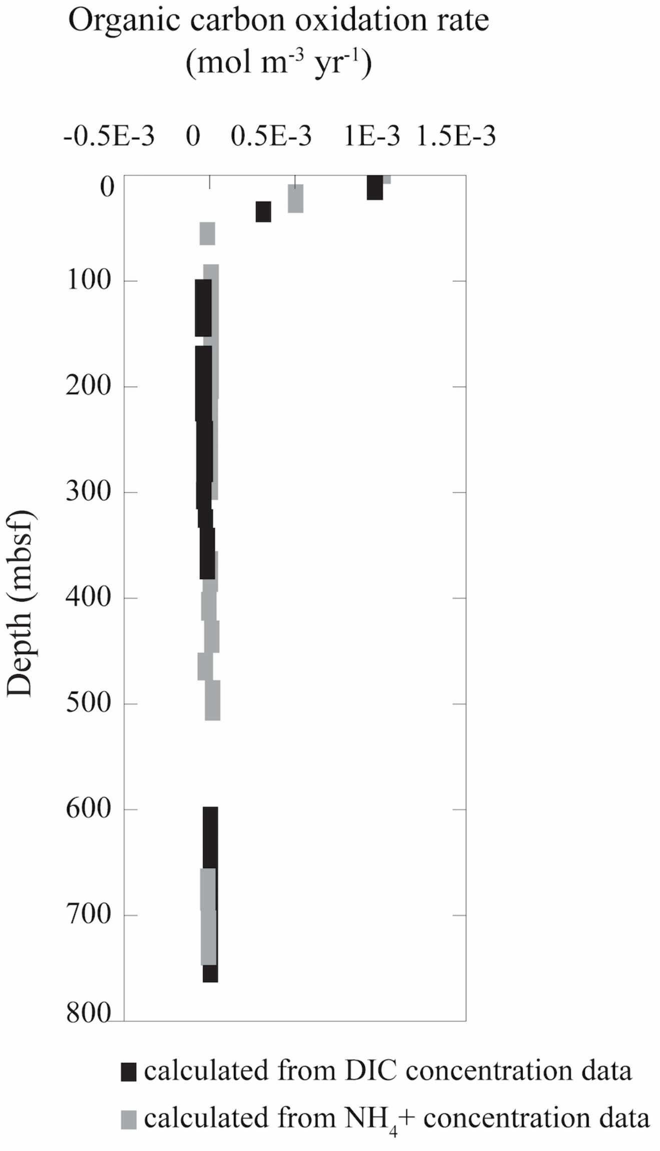

Figure S6. Vertical profiles of organic carbon oxidation calculated from DIC concentration data and from NH_4^+ concentration data.
